# Characterization of SHCBP1 to prognosis and immunological landscape in pan-cancer: novel insights to biomarker and therapeutic targets

**DOI:** 10.18632/aging.204591

**Published:** 2023-03-14

**Authors:** Fei Jiang, Yanlong Shi, Yue Wang, Chang Ge, Jun Zhu, Hanlu Fang, Yu Zhang, Yixiao Zhang, Haokun Jian, Tong Lei, Sheng Lan, Liyu Cao, Hongzhu Yu, Debao Fang

**Affiliations:** 1 Department of General Surgery, Fuyang Hospital of Anhui Medical University, Fuyang, Anhui, China; 2 Hepatopancreatobiliary Center, The Second Affiliated Hospital of Nanjing Medical University, Nanjing, Jiangsu, China; 3 Department of Pathology, Anhui Medical University, Hefei, Anhui, China; 4 Department of Oncology, Fuyang Hospital of Anhui Medical University, Fuyang, Anhui, China; 5 School of Basic Medicine, Hebei Medical University, Shijiazhuang, Hebei, China; 6 The Second Clinical Medical College, Lanzhou University, Lanzhou, Gansu, China; 7 The First Clinical College of Zhejiang Chinese Medical University, Hangzhou, Zhejiang, China; 8 School of Basic Medical Sciences, Xinxiang Medical University, Xinxiang, Henan, China; 9 The First Affiliated Hospital, Nanchang University, Nanchang, Jiangxi, China; 10 The Second Clinical College Clinical Medicine, Guangzhou Medical University, Guangzhou, Guangdong, China; 11 School of Basic Medical Sciences, Division of Life Sciences and Medicine, University of Science and Technology of China, Hefei, Anhui, China; 12 CAS Key Laboratory of Innate Immunity and Chronic Disease, Division of Life Sciences and Medicine, University of Science and Technology of China, Hefei, Anhui, China

**Keywords:** SHCBP1, prognosis, immune microenvironment, biomarker, pan-cancer

## Abstract

Background: Previous studies have revealed the significant roles of SHC SH2 domain-binding protein 1 (SHCBP1) in occurrence and progression of cancers, but there is no pan-cancer analysis of SHCBP1.

Methods: In this study, we explored the potential carcinogenic role of SHCBP1 across 33 tumors from the TCGA and GTEx databases. We investigated SHCBP1 expression, prognosis, genetic alterations, tumor mutational burden (TMB) score, microsatellite instability (MSI) and tumor microenvironment from TIMER2, GEPIA2, UALCAN and cBioPortal databases. Moreover, the cellular functions and potential mechanisms were evaluated by GO and KEGG analysis. Besides, the mRNA expression of SHCBP1 was examined using qRT-PCR assay in gastrointestinal cancers.

Results: SHCBP1 was significantly upregulated in various cancers, and apparent relationship existed between SHCBP1 and survival prognosis in patients. The TMB, MSI, and tumor microenvironment analysis indicated that SHCBP1 was closely related to immune checkpoints, immune targets, as well as CD4+ naive T cell, CD8+ T cell, and neutrophil. Moreover, the cellular functions of SHCBP1 were mainly in regulating cell cycle motor protein activity. In addition, we validated that SHCBP1 mRNA expression was over-expressed in gastrointestinal cancers.

Conclusions: This study was the first to systematically determine the prognostic value of SHCBP1, providing a forward-looking perspective on immunotherapy and cellular processes in pan-cancer.

## INTRODUCTION

Cancer is the leading proportion of chronic disease cases, with an increasing incidence, posing a significant threat to public health worldwide [1, 2]. Maintenance of genome stability is required for normal cellular metabolism, and loss of genome stability can contribute to tumorigenesis [3]. Thus, it is vital to develop a broad view of diagnosis and treatment encompassing many tumor types rather than focusing on histopathological diagnosis or molecular features to distinguish one tumor from another. Pan-cancer analysis aims to characterize and identify the commonalities and differences in genotypes and phenotypes of different cancers, which is essential to better understand complex oncologic lineages [4, 5]. Currently, it commonly utilizes high-throughput sequencing and public databases that possess abundant patient data further to elaborate the role of oncogenes [6].

Src homolog and collagen (SHC) are a key adaptor protein of cell surface receptors [7]. SHC SH2 domain-binding protein 1 (SHCBP1) is downstream of the SHC adaptor and binds to the SH2 domain of SHC [8]. SHCBP1 expression is highly expressed mainly in hyperplastic tissues and cells, suggesting that it is involved in physiological roles and pathological changes in the organism [9]. Emerging studies have demonstrated that SHCBP1 was up-regulated in several cancers, including breast [10], bladder [11], and gastric cancer [12]. Aberrant expression of SHCBP1 is involved in the occurrence, development, metastasis and prognosis of cancer, suggesting that SHCBP1 has potential value in terms of biomarkers and therapeutic targets [13]. In addition, as an intracellular signaling protein, SHCBP1 was associated with tumor growth, migration, and invasion via the mediation of several signaling pathways and regulation of the cell cycle, apoptosis, and differentiation [14]. However, a comprehensive pan-cancer study of SHCBP1 has yet to be reported in human cancers.

In this study, we explored and verified the potential roles of SHCBP1 in cancer development and progression via pan-cancer analysis, focusing on expression, prognosis, genetic alterations, protein phosphorylation, immune infiltration, and other relevant cellular processes. This comprehensive analysis highlights the multifaceted role of SHCBP1 and reveals its potential molecular mechanisms in generalized cancers.

## MATERIALS AND METHODS

### Gene expression analysis

We assessed SHCBP1 expression in tumors and normal tissues using the TIMER2 database with the following thresholds: *P*≤0.01, log2|fold change| (FC) = 1. (http://gepia2.cancer-pku.cn/#analysis) [15]. The GEPIA2 database was used to further investigate SHCBP1 expression in relation to prognosis and clinical stage, with “Pathological Stage Plot” and “Expression analysisBoxPlot” modules [16]. For survival, violin and box plots were used to visualize any significant differences. The total protein expression was obtained from the CPTAC of the UALCAN database (http://ualcan.path.uab.edu/analysis-prot.html) [17].

### Prognostic value analysis

The overall survival (OS) and disease-free survival (DFS) in the GEPIA2 database were applied to evaluate the prognostic value of SHCBP1 in different cancers. The survival map was obtained using the threshold 95% confidence intervals. The first progression (FP), disease-specific survival, post-progression survival (PPS), progression-free survival (PFS), and relapse-free survival (RFS) correlations were assessed in Kaplan–Meier plotter to determine the prognostic value in breast, lung, ovarian, GC, and liver cancer with the threshold value as: “auto select best cutoff” and log-rank P values [18].

### Genetic alteration analysis

We used the “TCGA PanCancer Atlas study” module to predict SHCBP1 alterations in the cBioPortal website with the following parameters: “Quick By Gene” and “SHCBP1” [19]. The altered species and sites were presented in histogram format. We further estimated the survival time between the SHCBP1-altered and -unaltered groups in the “Comparison/Survival” module.

### TMB, MSI, and tumor microenvironment analysis

Spearman correlation analyses were used to calculate the correlations of SHCBP1 expression in pan-cancers with tumor mutational burden (TMB) score, microsatellite instability (MSI), and two immune checkpoint pathway genes (inhibitory, stimulatory), with the results presented in bubble charts [20]. To further determine the relationship between SHHCBP1 and tumor immune, immune infiltration was analyzed using the CIBORSORT and XCELL algorithms. In addition, we also investigated the number of cancer-associated fibroblasts using the EPIC, MCPCOUNTER, and TIDE algorithms; the results are shown in a heatmap and scatter plot [21, 22].

### SHCBP1-related gene enrichment analysis

The “SHCBP1” and “Homo sapiens” parameters were used to acquire the top 50 SHCBP1-related altered genes using the STRING database (https://string-db.org/) with low confidence of 0.150. Then, based on the TCGA and GTEx datasets, the first 100 gene correlations were extracted from the “Gene_Corr” module of TIMER2. The intersection map of SHCBP1 was plotted using the Venn viewer Jvenn. The gene intersections were employed to generate a heatmap. In addition, according to the two gene datasets, we performed GO and KEGG enrichment analyses with the R packages “ClusterProfiler” and “ggplot2.” Cnetplot was used to show the molecular functions [23].

### Cell culture

Human colorectal cancer cells (SW480 and HCT116) and human normal colonic epithelial cells (NCM460) were purchased from Procell Life Science (Wuhan, China). Human gastric cancer cells (7901 and AGS), human gastric mucosa epithelial cells (GES), HCC cells (HepG2 and Huh), and human liver cells (LO2) were obtained from the School of Basic Medicine, Anhui Medical University. Cells were all cultured in DMEM supplemented with high glucose (HyClone) and 10% fetal bovine serum (VivaCell, Shanghai, China) at 5% CO2 and 37° C.

### Reverse transcription-quantitative polymerase chain reaction (RT-qPCR)

The total RNA content of tissues and cells was extracted using TRIzol reagent (Takara). According to the instructions, a PrimeScript™ kit (Takara) was used to perform reverse transcription. SYBR Green qPCR Mix was used to detect the relative expression of target genes based on the 2^−ΔΔCt^ method. The primer sequences used for the experiment were as follows: 5′-GCTACCGTGATA AACCAGGTTC-3′ (forward) and 5′-AGGCTCTGAATCGCT CATAGA-3′ (reverse).

### Statistical analysis

Student’s t-tests or one-way ANOVA analyses were employed to determine the significance of differential SHCBP1 expression in two groups or multiple groups, respectively. The OS, RFS, DFS, and DMFS were analyzed using the hazard ratios (HRs) and P values (or log-rank P values) between high- and low-risk groups or altered and unaltered groups. Spearman’s analysis was used to investigate the relationship between SHCBP1 expression and immune infiltration. Statistical significance was defined as *P<0.05*.

### Data availability statement

The datasets obtained and explored in this study are available in the TCGA, GEPIA2, UACLAN, cBioportal and TIMER2 databases.

## RESULTS

### SHCBP1 expression profiles in human cancers

**Figure 1:**
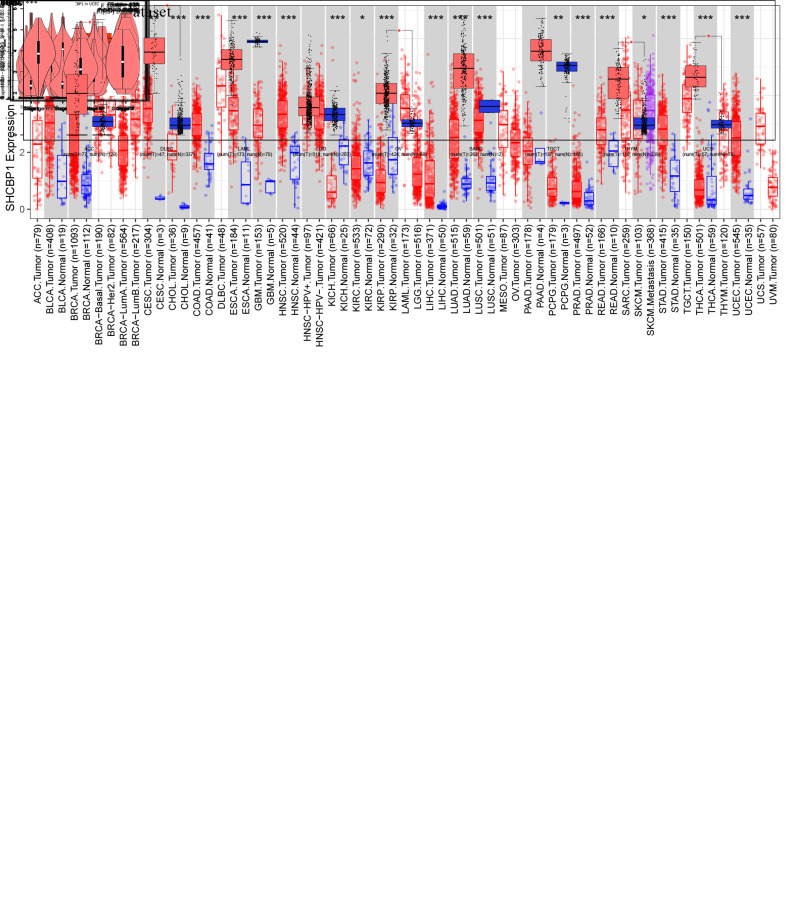
**Expression of SHCBP1 and its association with pathological stages.** (**A**) The expression of SHCBP1 was analyzed by using TIMER2 in TCGA dataset. (**B**) SHCBP1 expression in ACC, DLBC, LAML, LGG, OV, SARC, TGCT, THYM and UCS based on TCGA and GTEx dataset. (**C**) The expression of SHCBP1 protein in breast cancer, GBM, HNSC, LUAD, PAAD and UCEC based on UALCLAN database. (**D**) The association between SHCBP1 expression and pathological stages in certain cancers of GEPIA database. *P<0.05; **P<0.01; ***P<0.001.

Pan-cancer analysis was conducted to determine SHCBP1 expression levels in normal and tumor tissues using the TIMER and GTEx databases. In the TIMER2 database, compared with the normal tissues, the results showed elevated expression (*P*<0.001) of SHCBP1 in bladder urothelial carcinoma (BLCA), breast invasive carcinoma (BRCA), cholangiocarcinoma (CHOL), colon adenocarcinoma (COAD), esophageal carcinoma (ESCA), glioblastoma multiforme (GBM), head and neck squamous cell carcinoma (HNSC), kidney chromophobe carcinoma (KICH), kidney renal papillary cell carcinoma (KIRP), liver hepatocellular carcinoma (LIHC), lung adenocarcinoma (LUAD), lung squamous cell carcinoma (LUSC), prostate adenocarcinoma (PRAD), rectal adenocarcinoma (READ), stomach adenocarcinoma (STAD), thyroid carcinoma (THCA), and uterine corpus endometrial carcinoma (UCEC). In addition, SHCBP1 expression was elevated (*P*<0.01) in cervical squamous cell carcinoma, endocervical adenocarcinoma (CESC), and pheochromocytoma (*P*<0.01). Finally, SHCBP1 expression was elevated (*P*<0.05) in paraganglioma (PCPG), kidney renal clear cell carcinoma (KIRC), and skin cutaneous melanoma (SKCM). In total, 21 types of cancers showed elevated expression of this protein (Figure 1A). Because data on normal tissues are commonly missing in the TIMER2 database, we further explored SHCBP1 expression in the TCGA and GTEx databases of GEPIA. Utilizing this, we additionally found that SHCBP1 expression was higher (*P*<0.05) in adrenocortical carcinoma (ACC), lymphoid neoplasm diffuse large B-cell lymphoma (DLBC), ovarian serous cystadenocarcinoma (OV), thymoma (THYM), and uterine carcinosarcoma (UCS) than in the corresponding normal tissues. However, the expression of SHCBP1 showed no significant difference in pancreatic adenocarcinoma (PAAD), acute myeloid leukemia (AML), lower-grade glioma (LGG), sarcoma (SARC), or testicular germ cell tumors (TGCT) (*P*>0.05, Figure 1B). Next, we explored the expression of SHCBP1 protein in the CPTAC dataset. The results showed that SHCBP1 protein expression was over-expressed in BRCA, GBM, HNSC, LUAD, PAAD, and UCEC (*P*<0.001, Figure 1C). In addition, we analyzed the relationship between SHCBP1 expression and pathological stage in the GEPIA2 database. We found that SHCBP1 expression correlated with pathological stage in cases of ACC, BLCA, BRCA, KICH, KIRC, LIHC, LUAD, LUSC, KIRP, and TGCT (*P*<0.05, Figure 1D), while it did not correlate significantly with the stages of other cancers (Supplementary Figure 1).

### Analysis of the prognostic value of SHCBP1

**Figure 2:**
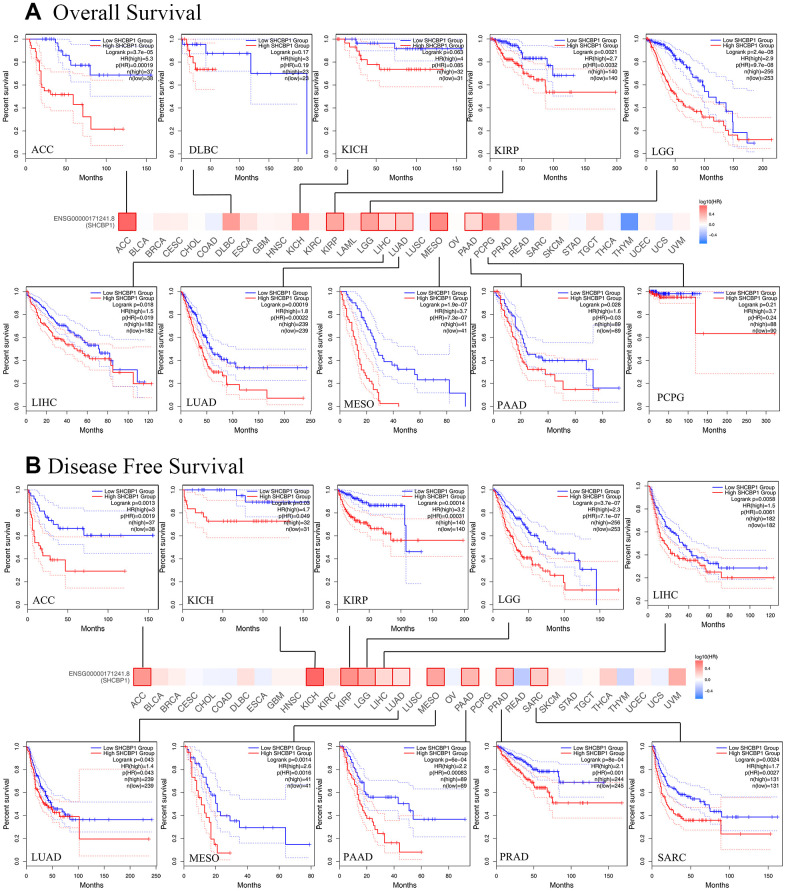
**The Kaplan-Meier survival curve was applied to investigate the prognosis of SHCBP1 expression in pan-cancer.** (**A**) Overall survival (**B**) Disease-free survival.

**Figure 3:**
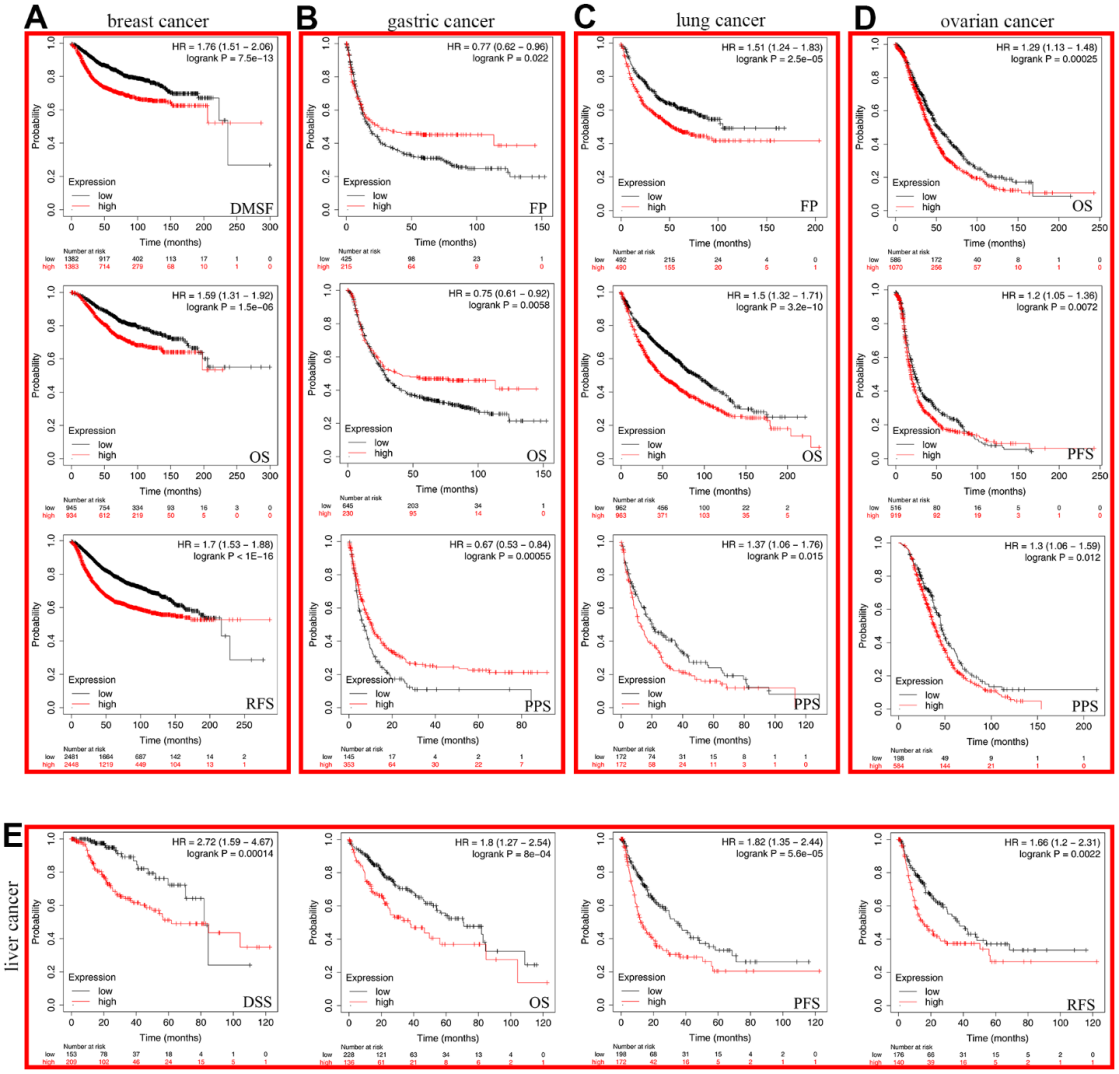
**The Kaplan-Meier plotter was used to analyze the prognostic role of cancer patients.** (**A**) breast cancer (**B**) gastric cancer (**C**) lung cancer (**D**) ovarian cancer (**E**) liver cancer.

To investigate the prognostic value SHCBP1 expression level, the “survival” module of the GEPIA2 database and Kaplan–Meier Plotter tool were used to explore the correlation between SHCBP1 expression and the prognosis of patients. In the GEPIA2 database, we found that the OS and DFS in the high-expression group were lower than those in the low-expression group in ACC, KIRP, LGG, LIHC, LUAD, MESO, and PAAD (*P*<0.05). Low expression of SHCBP1 was also associated with longer DFS in PRAD and SARC but not with higher OS (Figure 2A, 2B). Kaplan–Meier Plotter analysis indicated that low SHCBP1 expression correlated with better OS in breast (P=1.5e-06, HR=1.59 [1.31–1.92]), lung (P=3.2e-10, HR=1.5 [1.32–1.71]), ovarian (P=0.00025, HR=1.29 [1.13–1.48]), and liver (P=8e-04, HR=1.8 [1.27–2.54]) cancers (Figure 3A, 3C–3E). Conversely, high SHCBP1 expression was associated with longer FP (P=0.022, HR=0.77 [0.62–0.96]), OS (P=0.0058, HR=0.75 [0.61–0.92]), and PPS (P=0.00055, HR=0.67 [0.53–0.82]) in gastric cancer (Figure 3B). Accordingly, SHCBP1 may serve as a potential prognostic marker in certain cancers.

### Analysis of the effect of genetic alterations of SHCBP1

**Figure 4:**
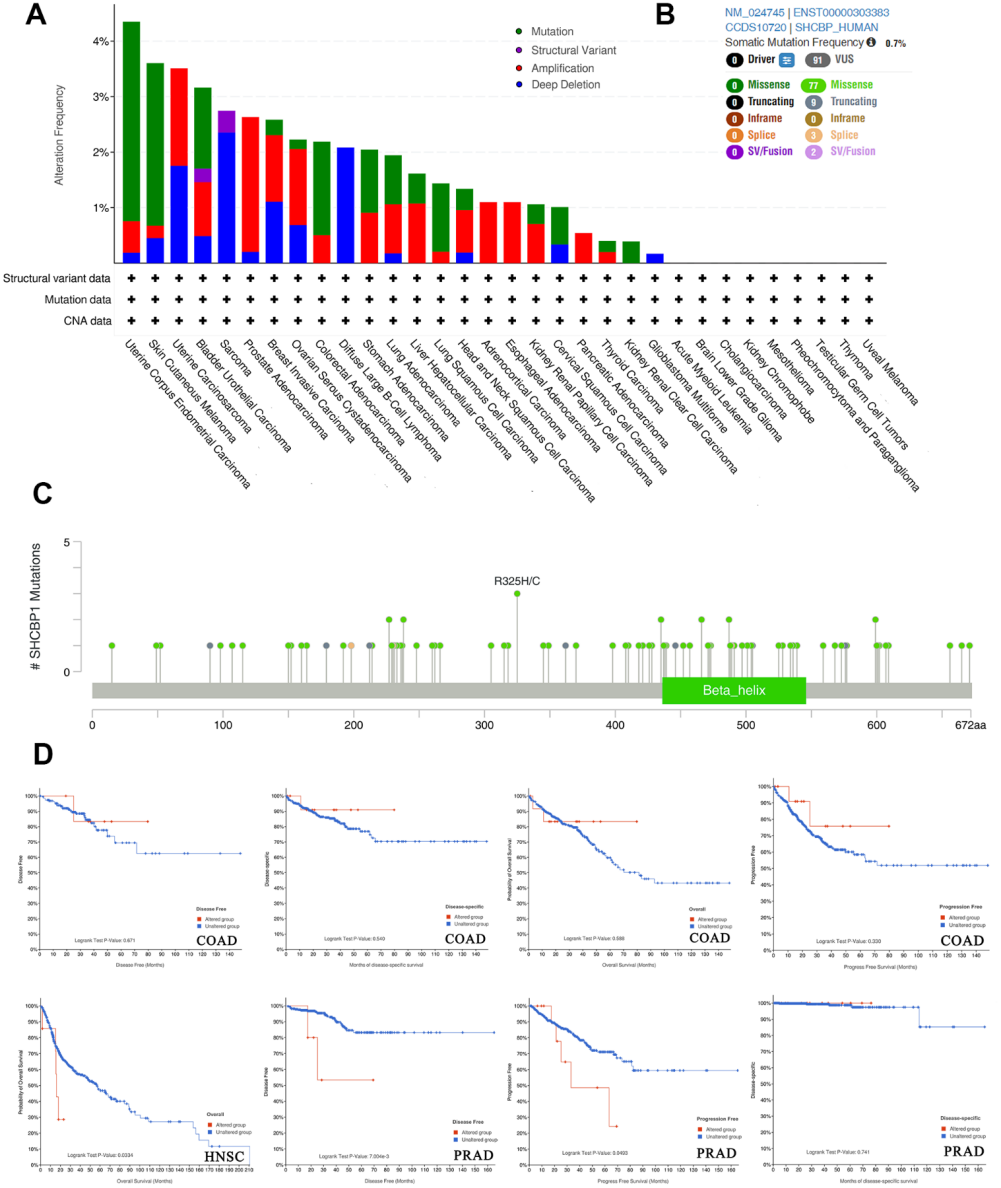
**Analysis of SHCBP1 alteration in pan-cancer.** (**A**) The histogram showed alteration frequency of SHCBP1 mutation types in cBioPortal database. (**B**) Summary of structural variation, mutations and copy number alterations of SHCBP1. (**C**) The mutation site with the highest alteration frequency (R325H/C) in SHCBP1. (**D**) The correlation between alteration status and prognosis in COAD, HNSC and PRAD patients.

Cancers arise through the accumulation of genetic mutations and epigenetic modifications. We conducted a genetic alteration analysis to study SHCBP1 mutations in human cancers. Amongst various human cancers, the alteration frequency of SHCBP1 (>5%) is highest in uterine corpus endometrial carcinoma (UCEC), in which “mutation” accounts for the primary proportion of alterations. PRAD showed the highest incidence of “amplification” alterations with a frequency of ~3% (Figure 4A). We then determined the different possible alterations at the R325H/C site, which included seventy-seven missense, nine truncating, and three splice and SV/Fusion alterations (Figure 4B, 4C). Further analysis showed no difference in the prognosis of COAD patients between the altered and unaltered groups. Patients with HNSC in the unaltered group presented with longer survival rates than those in the altered groups (*P*=0.033). In patients with PRAD, the unaltered group showed improved DFS (*P*=7.004e3) and PFS (*P*=0.049) but unchanged disease-specific survival (*P*=0.741, Figure 4D).

### Correlation analysis of SHCBP1 expression and immune checkpoints, TMB, and MSI

**Figure 5:**
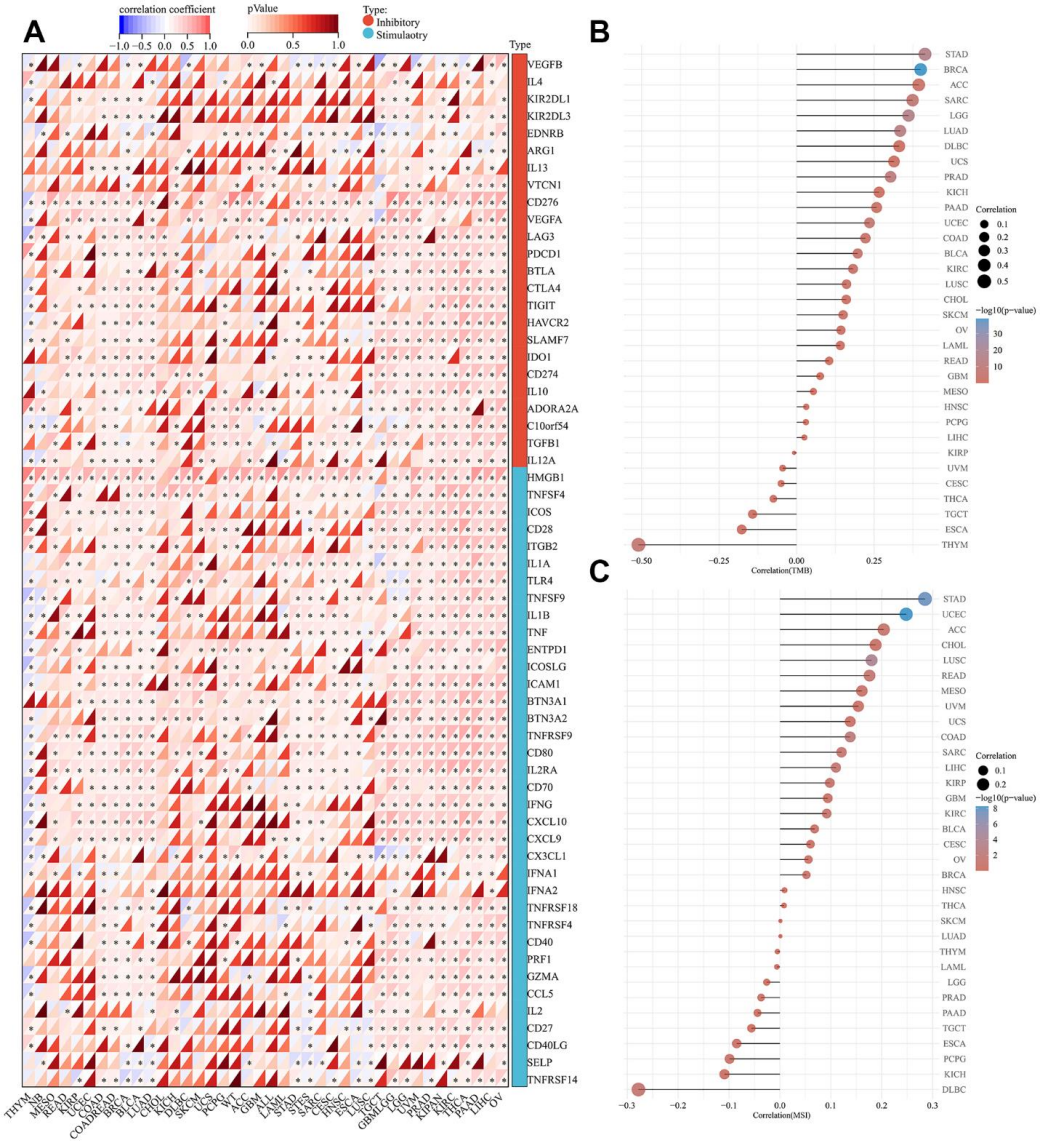
**Analysis of immunologic molecular, TMB, and MSI of SHCBP1 expression in pan-cancer.** (**A**) Correlation between 33 kinds of inhibitory, stimulatory and SHCBP1 expression. (**B**) Correlation between TMB and SHCBP1 expression. (**C**) Correlation between MSI and SHCBP1 expression. *P<0.05; **P<0.01; ***P<0.001.

We further estimated the role of SHCBP1 expression in modulating immune checkpoints. There were 24 immune inhibitors and 36 activators investigated across the various cancers. The expression of SHCBP1 was positively correlated with the expression of four inhibitors (KIR2DL3, IL13, PDCD1, and CD274) and five activators (HMGB1, ICOS, BTN3A1, BTN3A2, and PRF1) in the majority of tumors. However, in THYM, SHCBP1 expression was negatively correlated with the expression of all studied immune checkpoints except for KIR2DL3, EDNRB, ARG1, IL13, VTCN1, BTLA, IDO1, CD274, IL1O, TGFB1, IL1A, BTN3A1, IFNA2, PRF1, IL2, SELP, and TNFRSF14. CD276, VEGFA, TNFRSF9, TNFSF4, and HMGB1 play important immune roles in various tumors (Figure 5A). Gene mutations are an important inducing factor in tumorigenesis [24]. TMB and MSI can cause polymorphisms in immunogenic peptides and microsatellite sequence length, respectively, leading to genomic instability [25]. The results revealed that TMB was positively associated with SHCBP1 expression in 18 types of cancers while negatively correlated in THYM and ESCA (Figure 5B). There is also likely a close correlation between SHCBP1 expression and MSI in UCEC, STAD, LUSC, COAD, READ, and LIHC (Figure 5C).

### Correlation analysis between SHCBP1 expression and immunity

**Figure 6:**
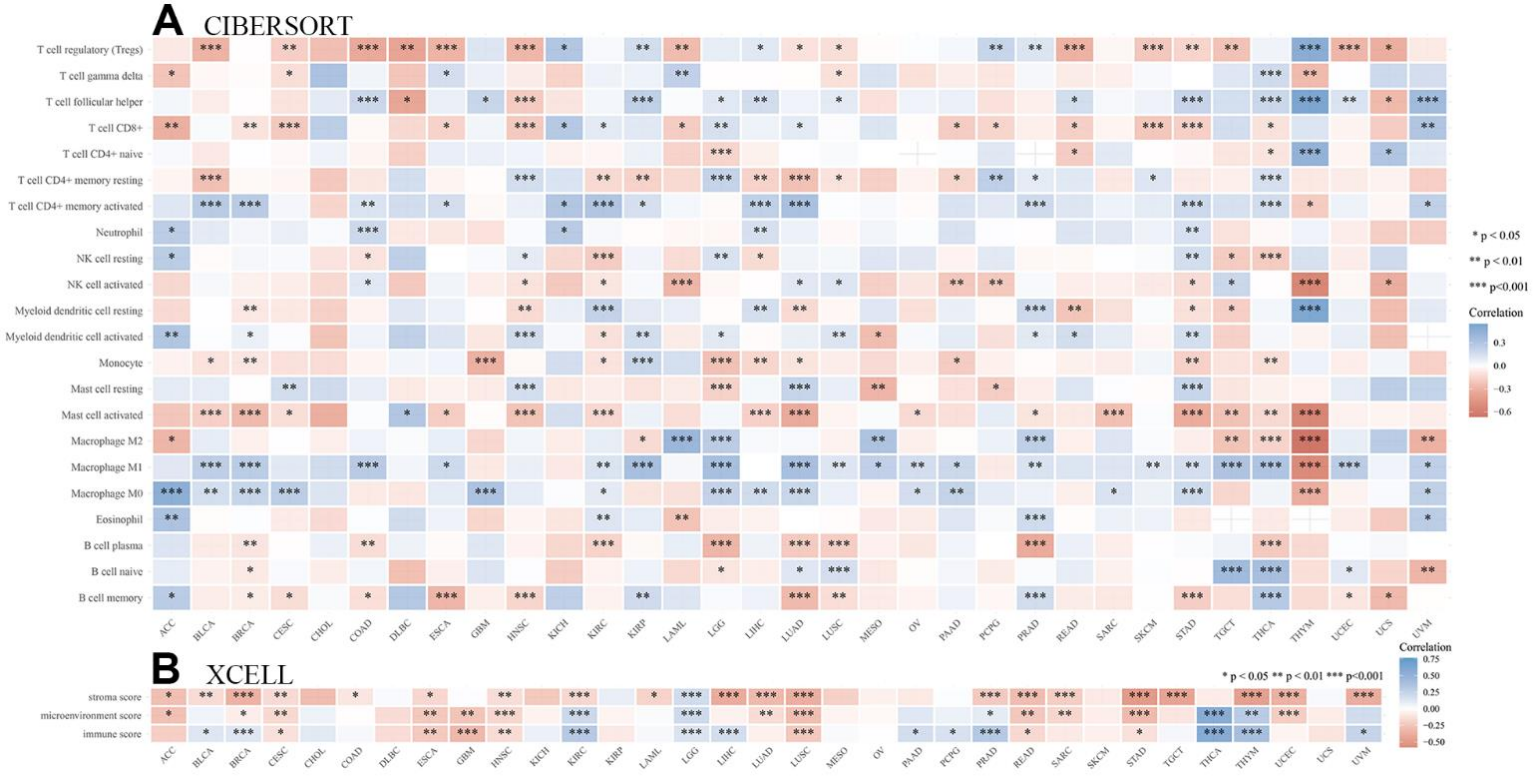
**Analysis of the immunity role of SHCBP1 expression in pan-cancer.** (**A**) The correlations between SHCBP1 expression and immune infiltration cells. (**B**) The correlations between SHCBP1 expression and stromal score, microenvironment score and immune score. *P<0.05; **P<0.01, ***P<0.001.

**Figure 7:**
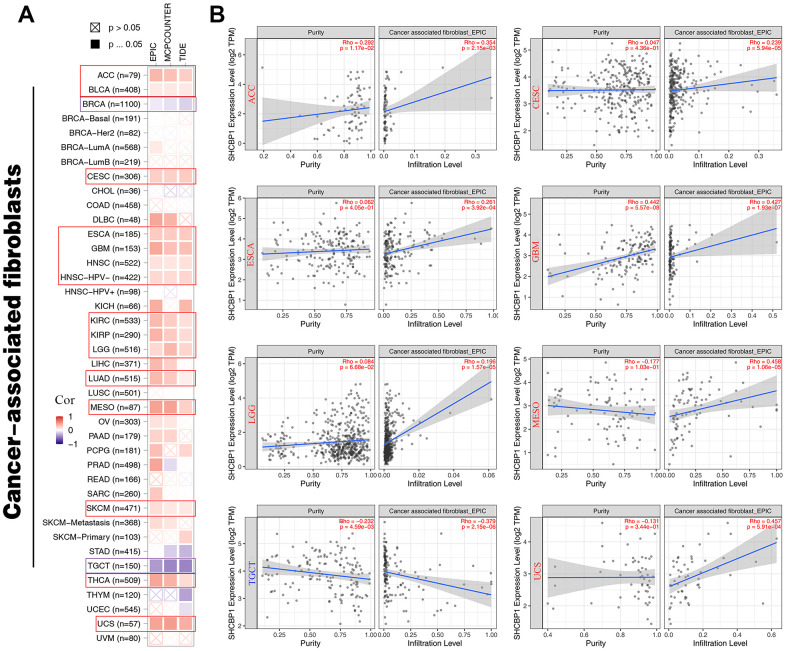
**The relationship between SHCBP1 expression and cancer-associated fibroblasts in pan-cancer.** (**A**) The EPIC, MCPCUNTER, and TIDE algorithms was conducted to investigate cancer-associated fibroblasts by heatmap. (**B**) The purity and infiltration level were presented in scatter plots on ACC, CESC, ESCA, GBM, LGG, MESO, TGCT and UCS.

The mutation rate of tumors is closely associated with immune cell function [26, 27]. Therefore, we identified the relationships between immune infiltration level and SHCBP1 expression using the CIBERSORT and XCELL algorithms. In Figure 6A, the results demonstrated that SHCBP1 expression was positively correlated with CD8+ T cell infiltration in ACC, BRCA, CESC, ESCA, HNSC, AML, PAAD, PCPG, READ, SKCM, STAD, and THCA, while negatively correlated in KICH, KIRP, LGG, LUAD, and UVM. SHCBP1 expression was also related to CD4+ naive T cell infiltration in LGG, READ, THCA, THYM, and UCS, and neutrophil infiltration in ACC, COAD, KICH, LIHC, and STAD. Moreover, we investigated the relationships between SHCBP1 expression and stroma, microenvironment, and immune scores. We found that stroma score was closely correlated to SHCBP1 expression in most cancers except in DLBC, GBM, KICH, KIRP, MESO, OV, PAAD, PCPG, SKCM, THCA, and UCS. LGG was the only cancer type that showed a negative correlation with SHCBP1 expression (Figure 6B). Interestingly, the microenvironment and immune scores showed similar correlations with SHCBP1 expression in pan-cancers other than in BRCA. In addition, EPIC, MCPCOUNTER, and TIDE algorithms were used to assess the association between cancer-associated fibroblast infiltration and SHCBP1 expression in 33 kinds of cancers. As shown in Figure 7A, 7B, SHCBP1 expression had positive correlations with cancer-associated fibroblast infiltration in ACC, BLCA, CESC, ESCA, GBM, HNSC, HNSC-HPV-, KIRC, KIRP, LGG, LUAD, MESO, SKCM, THCA, and UCS, and negative correlations in BRCA and TGCT. We visualized these correlations using scatter plots for ACC, CESC, ESCA, GBM, LGG, MESO, TGCT, and UCS.

### SHCBP1-related gene enrichment analysis

**Figure 8:**
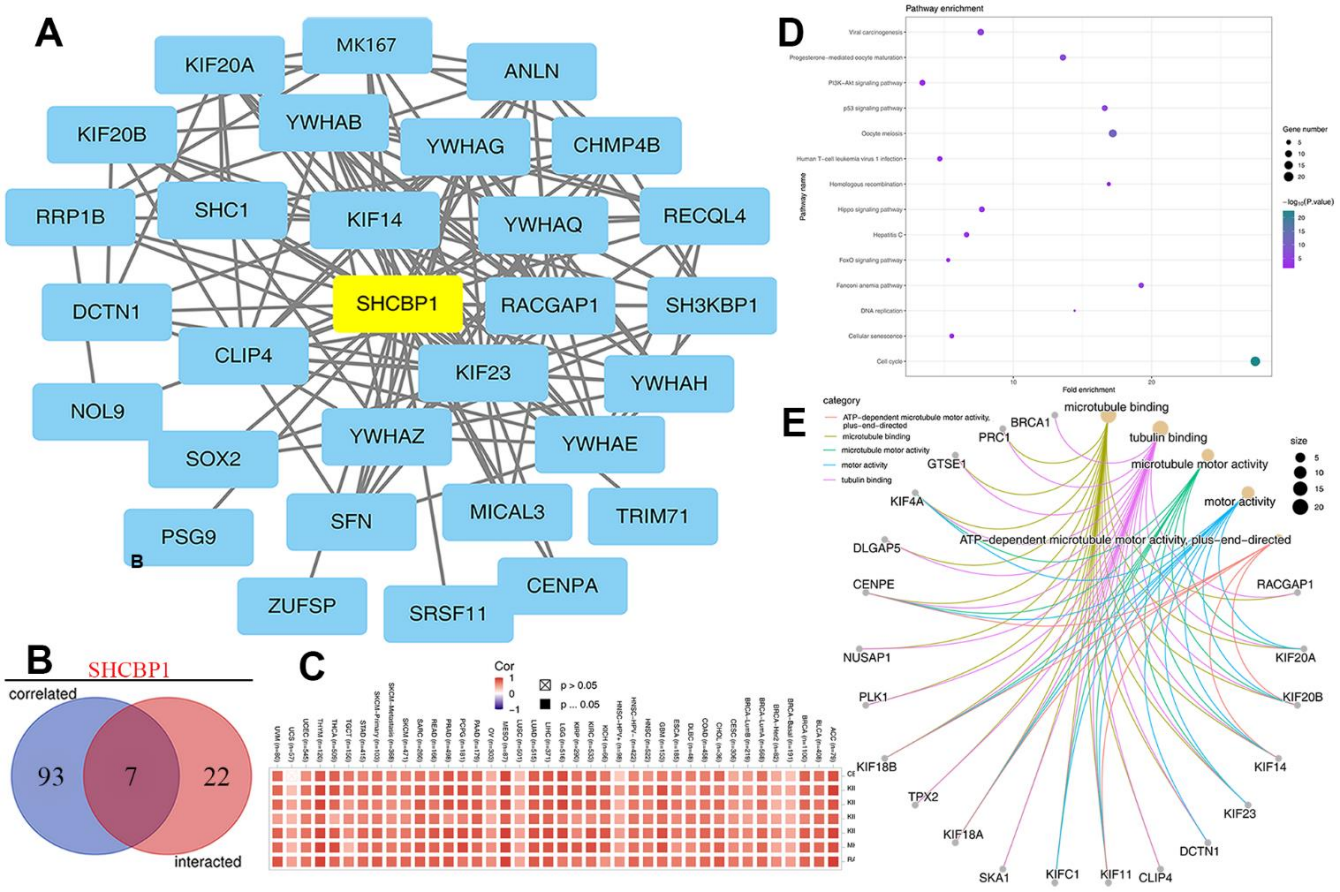
**Analysis of SHCBP1 potential cellular functions.** (**A**) The interaction network of first 50 SHCBP1-binding proteins in STING database. (**B**) The Venn diagram identified 7 genes (CENPA, MKI67, RACGAP1, KIF20A, KIF20B, KIF14, KIF23) based on SHCBP1-binding and related genes. (**C**) Heatmap analysis of the expression of 7 genes in pan-cancer. (**D**) Analysis of pathway enrichment. (**E**) The molecular function of GO analysis was investigated by Cnetplot.

To further determine the role of SHCBP1, we employed enrichment analysis based on SHCBP1-binding proteins and co-expressed genes using the STRING and GEPIA2 databases. The analysis of the STRING database generated 50 SHCBP1-binding genes using data from previous experiments, which were plotted in interaction networks (Figure 8A). Then, we obtained SHCBP1’s top 100 co-expressed genes using the “correlation analysis” module of GEPIA2. According to the determined SHCBP1 binding genes, we identified seven genes using a Venn diagram for further exploration, including CENPA, MKI67, RACGAP1, KIF20A, KIF20B, KIF14, and KIF23 (Figure 8B). We constructed a heatmap to display the correlations between SHCBP1 expression and the aforementioned seven genes in pan-cancers (Figure 8C). In addition, KEGG enrichment analysis showed that SHCBP1 was mainly involved in cell cycle regulation, Fanconi anemia, oocyte meiosis, viral carcinogenesis, p53 signaling, and PI3K-Akt signaling (Figure 8D). Moreover, the MF of GO analysis indicated that the primary function of SHCBP1-related genes was in tubulin and microtubule-binding, followed by microtubule motor activity, motor protein activity, and plus-end-directed ATP-dependent microtubule motor activity (Figure 8E).

### SHCBP1 expression validation using cancer cell lines

**Figure 9:**
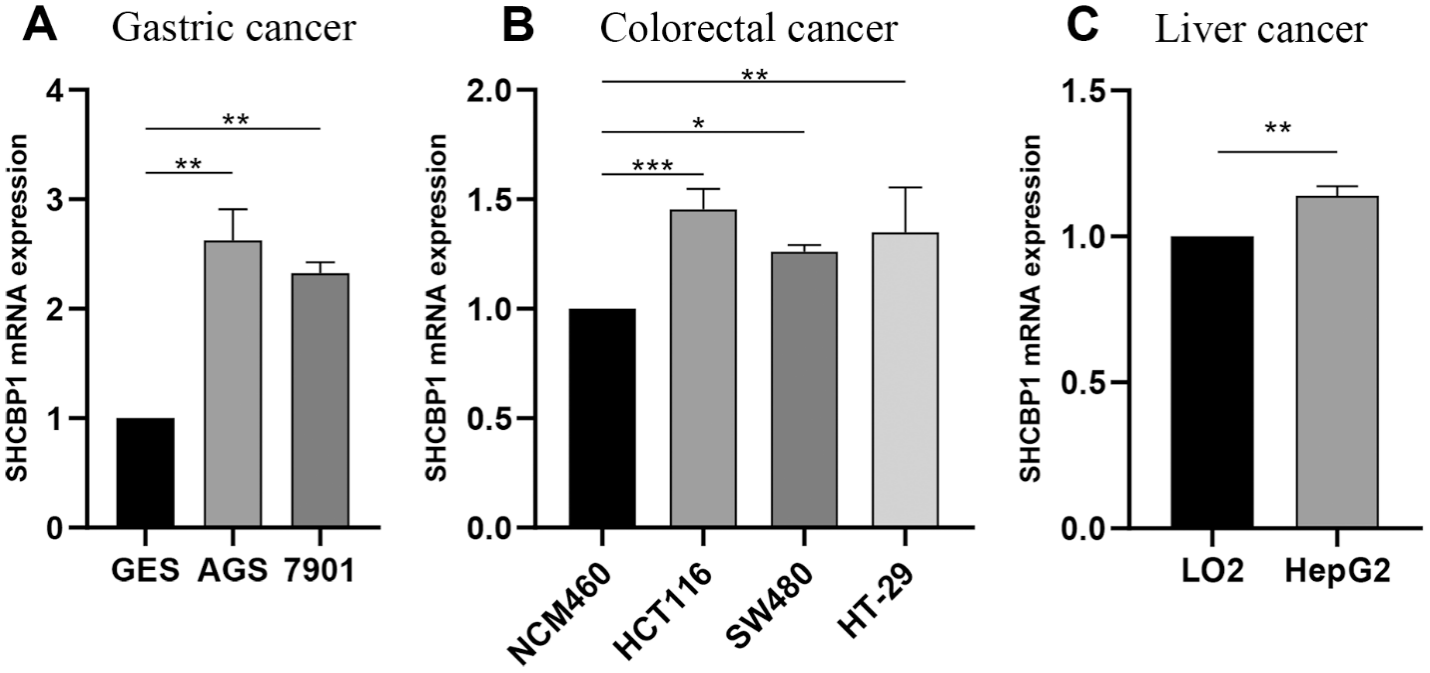
**Experimental verification of SHCBP1 mRNA expression by qRT-PCR analysis in various cancers.** (**A**) Gastric cancer (**B**) Colorectal cancer (**C**) Liver cancer. *P<0.05; **P<0.01; ***P<0.001.

To validate the above results, we detected the expression of SHCBP1 in GC, CRC, and HCC cell lines. The experimental results indicated that SHCBP1 expression was upregulated in GC (AGS and 7901 cell lines), CRC (SW480 and HCT116 cell lines), HCC (HepG2 cell line) cells compared to their corresponding control groups (Figure 9A–9C).

## DISCUSSION

SHCBP1 involved in various physiological and pathological processes, such as cytokinesis during mitosis and meiosis [8]. Recently, a growing number of studies have shown that SHCBP1 promotes tumorigenesis and progression of human tumors and is closely related to cancer prognosis. The stronger the expression of SHCBP1 protein in breast cancer tissues, the later the clinical stage and the shorter the survival period of the patients [10]. The expression of SHCBP1 was associated with lymph node metastasis in glioma, and patients with over-expressed SHCBP1 had a poor prognosis [28]. Moreover, the knockout of SHCBP1 reduced the migration and invasion ability in EGF-induced bladder cancer cells [11]. In addition, SHCBP1 plays an important role in FGF, NF-κB, MAPK/ERK, PI3K/AKT, and TGF-β1/Smad signaling, as well as participating in T cell development and downstream transduction regulation [7]. However, despite SHCBP1 being extensively studied *in vitro* previously, SHCBP1’s role in pan-cancer remains unclear.

In this study, we analyzed the expression of SHCBP1 in 33 tumor types, and SHCBP1 expression was elevated in most of these types except for PAAD, AML, LGG, SARC, and TGCT when compared with the corresponding normal tissues. Moreover, our experimental results verified that SHCBP1 expression was upregulated in the liver, gastric, and colorectal cancer cell lines, which was consistent with public database information. In addition, we found that SHCBP1 protein expression was also upregulated in BRCA, GBM, HNSC, LUAD, PAAD, and UCEC. High expression of SHCBP1 was correlated with tumor pathological stage in several cancers. In addition, the GEPIA database analysis showed that upregulated SHCBP1 was negatively correlated with patients' OS and DFS in ACC, KIRP, LGG, LIHC, LUAD, MESO, and PAAD. However, elevated SHCBP1 was correlated with a better prognosis in patients with GC. This is inconsistent with previous studies, which may be due to different analysis methods for different databases and studies. In general, SHCBP1 can promote tumor growth and invasion in GC by regulating the CDK4-cyclin D1 cascade and caspase-3 and caspase-PARP-dependent apoptosis pathways [12]. Our results indicate that elevated SHCBP1 expression is associated with unfavorable prognosis in different cancers and may be a promising biomarker for accurate diagnosis and treatment.

The SHCBP1 gene is located in a region of chromosome 16q11.2 [29]. In the cBioPortal database, there are several reported alterations of SHCBP1 in pan-cancers, with the most prominent types being “mutation” and “amplification.” We identified that prognosis in the unaltered group was better than that in the altered group. As a key binding protein of cell surface receptors, mutation and amplification of SHCBP1 may lead to abnormal binding of SHC proteins, resulting in physiological and metabolic dysfunction. Importantly, detecting SHCBP1 gene mutations will be valuable for improved prognosis and targeted therapy efficacy.

Tumor immune escape is often due to the disruption of immune cell-mediated checkpoint pathways by tumor cells [30, 31]. However, this means that checkpoint components present possible targets for immunotherapy. Immune checkpoint blocking therapies increase the aggressiveness of the host immune system to tumor cells and potentiate programmed death receptors and their ligands [32]. The most commonly used biomarkers for predicting ICI are MSI, TMB, and PD-L1 [33, 34]. In our study, SHCBP1 expression was positively correlated with four immune inhibitors (KIR2DL3, IL13, PDCD1, and CD274) and five activators (HMGB1, ICOS, BTN3A1, BTN3A2, and PRF1) in the majority of tumors. Moreover, TMB was positively associated with SHCBP1 expression in 18 types of cancers. SHCBP1 expression was also correlated with MSI in UCEC, STAD, LUSC, COAD, READ, and LIHC. Additionally, to investigate SHCBP1’s influence on the tumor microenvironment, the relationship between SHCBP1 expression and immune cell infiltration was investigated in different cancers. Using a combination of CIBERSORT and XCELL algorithms, we found that SHCBP1 expression was positively correlated with CD8+ T cell infiltration in 12 types of cancer but negatively in 5 kinds of cancer. SHCBP1 expression was also related to CD4+ naive T cell infiltration (LGG, READ, THCA, THYM, and UCS) and neutrophil infiltration (ACC, COAD, KICH, LIHC, and STAD). Moreover, the stroma, microenvironment, and immune scores were closely correlated with SHCBP1 expression. In addition, cancer-associated fibroblasts cells may play a vital role in modulating SHCBP1 expression. These results demonstrate that targeting SHCBP1 can influence tumor immunotherapy. So far, little is known about the role of SHCBP1 in the tumor immune microenvironment, which is worthy of further investigation.

This study had several limitations. Firstly, different database algorithms may cause errors in the analysis [35]. Secondly, there is a need for prospective studies in order to validate SHCBP1’s prognostic value. Thirdly, although we have verified the expression of SHCBP1 in several cancers, the exact molecular mechanisms underlying its mechanisms in cancer still require investigation.

## CONCLUSIONS

This study is the first to systemically explore the roles of SHCBP1 in pan-cancer. SHCBP1 expression was associated with clinical prognosis, genetic alterations, immune checkpoint expression, TMB, MSI, immune cell infiltration, and cellular processes, which provided a forward-looking view for effective tumor diagnosis and treatment. Future studies should further illuminate the potential mechanisms by which SHCBP1 modulates the immune microenvironment and lay a solid foundation for therapeutic target.

## Supplementary Material

Supplementary Figure 1
