# Supplementary material for: Characterization of SHCBP1 to prognosis and immunological landscape in pan-cancer: novel insights to biomarker and therapeutic targets
Source: Aging (Albany NY). 2023 Mar 14;15(6):2066–81. doi: 10.18632/aging.204591 (PMC10085602; doi:10.18632/aging.204591)
Supplement: Supplementary Figure 1 [file aging-15-204591-s001.pdf]

SUPPLEMENTARY FIGURE

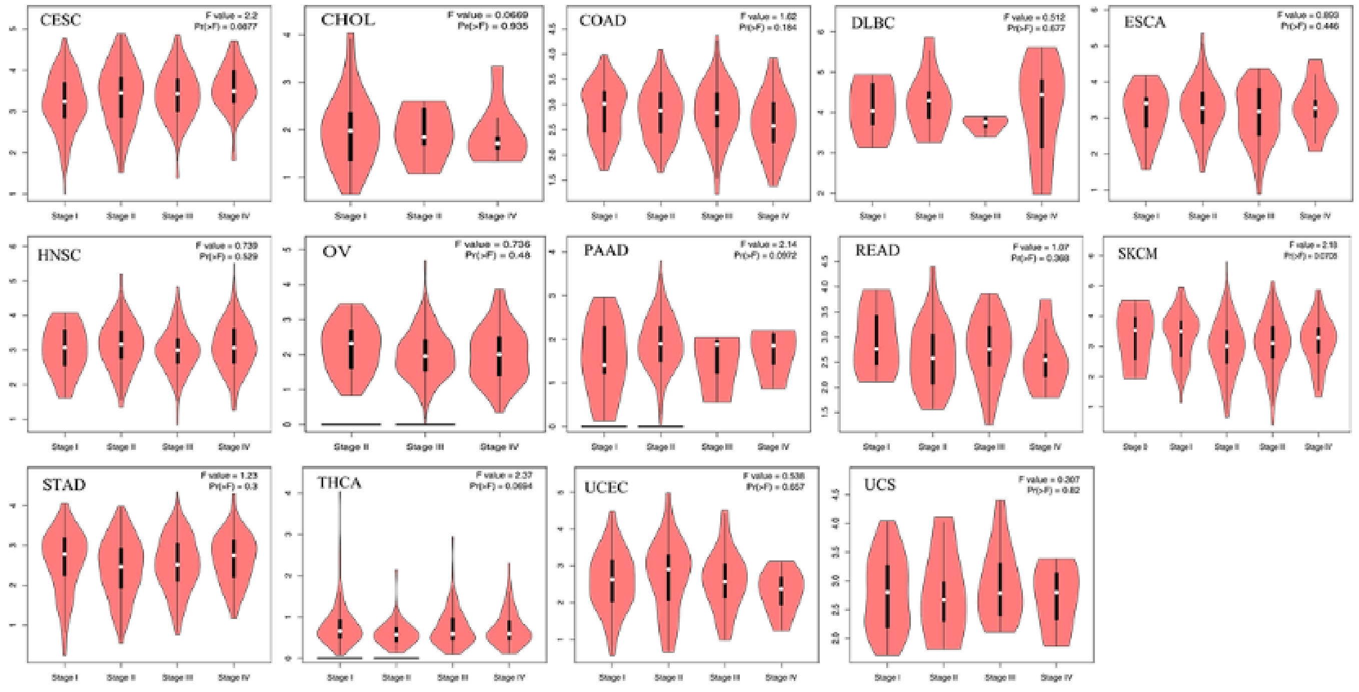

Supplementary Figure 1. The association between SHCBP1 expression and pathological stages in other cancers of GEPIA database.
